# Kidney-alone transplantation in prior SLK candidates: a national registry analysis

**DOI:** 10.3389/ti.2026.16834

**Published:** 2026-08-27

**Authors:** Beat Moeckli, Kevin B. Harris, Byron Smith, David Pereyra, Xiomara Benavides, Jody Olson, Alexander R. Cortez, Timucin Taner, Julie K. Heimbach, Douglas A. Simonetto, Samy M. Riad

**Affiliations:** 1 Department of Surgery, University of Geneva, Geneva, Switzerland; 2 Division of Gastroenterology, Department of Medicine, Henry Ford Health, Detroit, MI, United States; 3 Transplant Center, Mayo Clinic Minnesota, Rochester, MN, United States

**Keywords:** chronic liver diseases, cirrhosis, end stage renal disease, graft survival, kidney transplantation, simultaneous liver-kidney transplantation, compensated cirrhosis

## Abstract

Outcomes of kidney-alone transplantation (KAT) in former simultaneous liver-kidney (SLK) transplant candidates after liver waitlist delisting are not well defined. Using Scientific Registry of Transplant Recipients (SRTR) data, we identified adult recipients who underwent KAT between 2005 and 2025 after prior SLK listing (RLD-KAT), which was matched 1:5 to primary KAT recipients without liver disease by age, transplant year, and diabetes status. Patient and graft survival were evaluated using the Kaplan-Meier method and multivariable Cox proportional hazards models. We identified 163 RLD-KAT recipients matched to 815 controls. Unadjusted Kaplan-Meier analyses demonstrated lower patient survival in RLD-KAT (p = 0.020), whereas death-censored and overall graft survival were similar (p > 0.05). In multivariable models, death-censored graft survival (HR 0.89; 95% CI 0.51–1.53), overall graft survival (HR 1.00; 95% CI 0.73–1.36), and patient survival (HR 1.07; 95% CI 0.76–1.5) did not differ significantly. Six RLD-KAT recipients (3.7%) subsequently underwent liver transplantation. Kidney-alone transplantation in prior SLK candidates was associated with acceptable patient and graft outcomes and a low incidence of subsequent liver transplantation.

## Graphical Abstract

**Figure:**
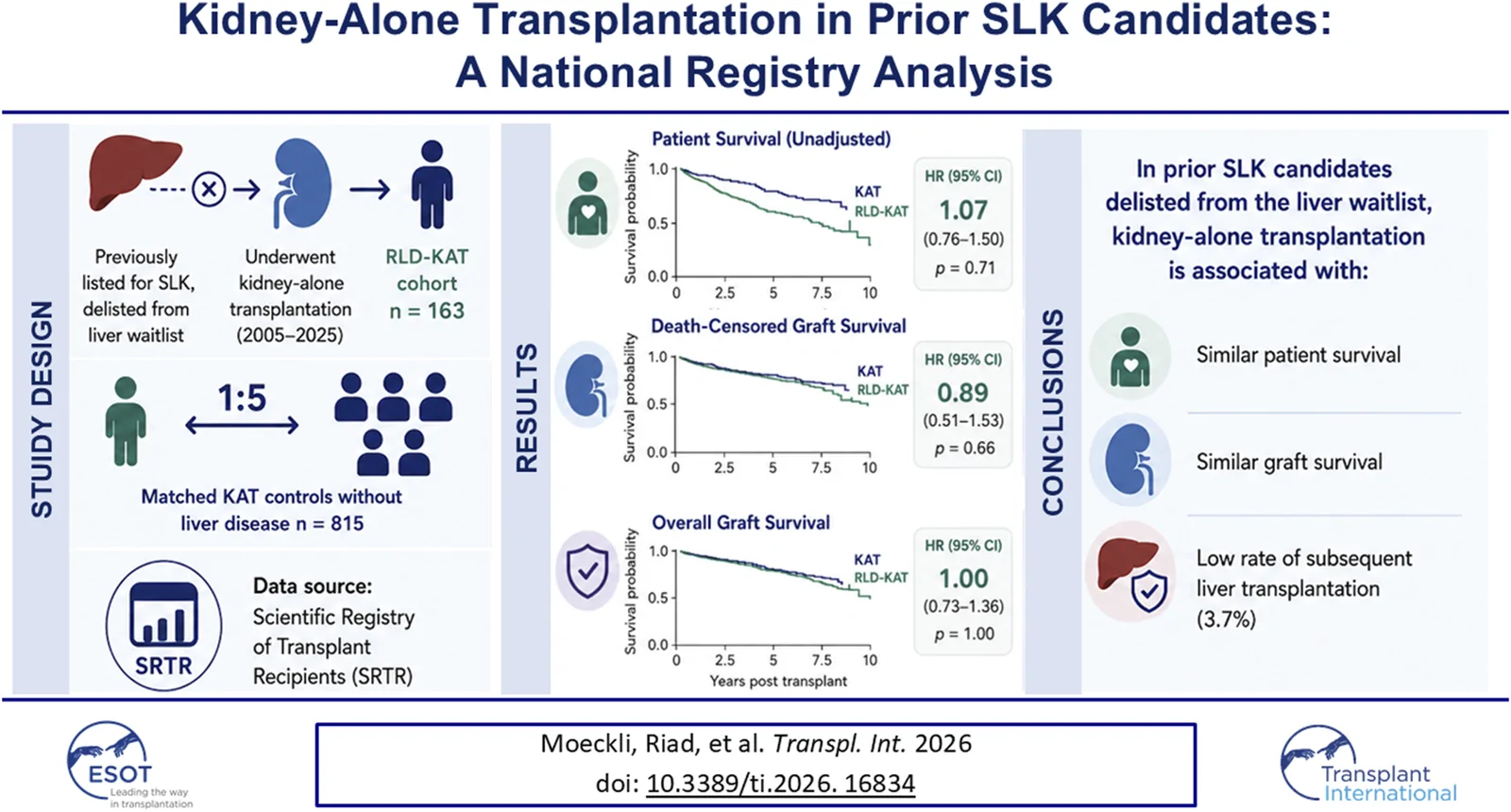

## Introduction

Chronic kidney disease (CKD) is increasingly common among liver transplant candidates, driven by shifts in liver disease epidemiology and rising rates of concurrent renal dysfunction [1, 2]. In the United States, alcohol-associated liver disease and metabolic dysfunction–associated steatohepatitis (MASH) have emerged as the leading indications for liver transplantation [3], both of which are strongly associated with CKD [2, 4]. This evolving landscape necessitates more nuanced decisions regarding organ allocation, particularly in balancing the use of simultaneous liver–kidney transplantation (SLK) versus kidney-alone transplantation (KAT).

In patients with compensated cirrhosis, early SLK listing practices were shaped by concerns that kidney transplantation alone could precipitate hepatic decompensation, leading to conservative strategies that favored dual-organ allocation [5–7]. This caution remains evident in the kidney transplant community, where hesitancy persists in offering KAT to patients with compensated cirrhosis, even when synthetic liver function appears preserved [8, 9]. Central concerns include the risk of post-transplant liver decompensation, progression to end-stage liver disease, and inadequate assessment of portal hypertension or ongoing hepatic injury [10].

In the United States, candidates are considered for simultaneous liver–kidney transplantation (SLK) only after meeting accepted indications for liver transplantation while also satisfying kidney-specific eligibility criteria established by the Organ Procurement and Transplantation Network (OPTN). Current OPTN policy defines SLK eligibility primarily by the severity and duration of kidney dysfunction, including prolonged dialysis dependence or sustained reductions in estimated glomerular filtration rate (eGFR), rather than by standardized measures of liver disease reversibility [11, 12].

While these criteria improved consistency in SLK allocation, they do not directly address the dynamic nature of liver disease or changes in transplant candidacy over time. Consequently, transplant decision-making often shifts to the kidney transplant community when candidates initially listed for SLK reach a point where liver transplantation is no longer pursued yet continue to require kidney transplantation. In this setting, uncertainty remains regarding the safety of proceeding with kidney-alone transplantation (KAT), particularly in the absence of standardized measures of hepatic stability or objective assessment of portal hypertension. Consequently, centers may vary in their willingness to proceed with KAT. Although several single-center studies suggest that KAT may be feasible in carefully selected patients, long-term outcomes in this unique population remain poorly characterized [13–15].

Recently, our group examined national waitlist outcomes among more than 15,000 candidates listed for SLK transplantation using the SRTR. That study demonstrated that kidney-alone transplantation following SLK listing is uncommon, occurring in approximately 1%–1.5% of candidates, while approximately 15% were removed from the waitlist because transplantation was no longer considered necessary. These findings highlight the dynamic nature of transplant candidacy and suggest that a small but clinically important subgroup of candidates may ultimately proceed with kidney-alone transplantation. However, the long-term post-transplant outcomes of these recipients remain unknown [16].

This study leverages national SRTR data to evaluate outcomes in recipients who were initially listed for SLK but ultimately received KAT following liver waitlist delisting. By comparing these recipients with matched kidney-alone transplant recipients without liver disease, we characterize patient and graft survival, evaluate the subsequent need for liver transplantation, and provide evidence to inform transplant decision-making in this uncommon but clinically important population.

## Materials and methods

### Data source

This study used data from the Scientific Registry of Transplant Recipients (SRTR). The SRTR data system includes data on all donor, wait-listed candidates, and transplant recipients in the US, submitted by the members of the Organ Procurement and Transplantation Network (OPTN). The Health Resources and Services Administration (HRSA), U.S. Department of Health and Human Services provides oversight to the activities of the OPTN and SRTR contractors.

Data access for this study was obtained through the SRTR Data Access Portal under approved data-use agreements, and analyses were conducted in compliance with applicable ethical guidelines and regulatory requirements. This study was deemed exempt by the Mayo Clinic internal review board.

### Study population

We analyzed all adult (≥18 years) kidney-alone transplant (KAT) recipients in the Scientific Registry of Transplant Recipients (SRTR) between 2005 and 2025. Recipients were identified using linked liver and kidney transplant records within the Scientific Registry of Transplant Recipients (SRTR). The study cohort consisted of adults with chronic liver disease who were initially listed for simultaneous liver–kidney transplantation (SLK) before their first kidney transplant, had no history of prior liver or kidney transplantation, and ultimately underwent kidney-alone transplantation (KAT). To preserve the temporal sequence between liver listing and kidney transplantation, only liver waitlist registrations preceding the first kidney transplant were considered.

Because SRTR does not capture physiologic markers of hepatic recovery, the cohort was characterized using liver waitlist status before kidney transplantation. Throughout the manuscript, this administratively defined cohort is referred to as recompensated liver disease kidney-alone transplantation (RLD-KAT) for simplicity. The acronym reflects the clinical pathway leading to kidney-alone transplantation rather than direct biologic evidence of hepatic recompensation. RLD-KAT recipients were then matched in a 1:5 ratio to primary kidney-alone transplant recipients without prior liver waitlist registration (hereafter referred to as the KAT cohort) using recipient age (±5 years), kidney transplant year, and diabetes status.

### Outcomes of interest

The primary outcome of the study was the patient and graft survival. Death-censored graft survival is defined as time from transplant to graft failure with death treated as a censoring event. Overall graft survival is defined as time from transplant to graft loss or patient death. Recipient survival is defined as time from transplant to death from any cause. Secondary outcomes included subsequent liver transplantation, repeat kidney transplantation, and cause-specific mortality where available.

### Variables and definitions

Recipient characteristics include age, sex, race/ethnicity, body mass index (BMI), diabetes status, hepatitis B and C status, dialysis status and duration, serum creatinine, steroid maintenance, induction therapy, and other relevant comorbidities. Donor characteristics included donor age and sex, BMI, donor type, kidney donor risk index (KDRI) and human leukocyte antigen (HLA) mismatch. Transplant characteristics include local organ availability, kidney cold ischemia time, and induction regimens.

For the RLD-KAT cohort, additional liver-related variables were collected to characterize the clinical trajectory after initial SLK listing, including liver disease etiology, MELD score at SLK listing, INR, ascites, encephalopathy, prior TIPS, liver waitlist removal or inactivation, documented liver waitlist removal reason, and the interval between liver waitlist status change and kidney transplantation, when available.

### Statistical analyses

Recipients in the RLD-KAT cohort were matched to primary KAT recipients in a 1:5 ratio based on age (±5 years), transplant year and diabetes status to ensure demographic comparability and reduce confounding by secular trends in transplant practice.

Descriptive statistics were used to summarize baseline characteristics. Continuous variables are presented as means with standard deviations or medians with interquartile ranges, depending on distribution. Categorical variables are summarized as counts and percentages. Group comparisons used chi-square or Fisher’s exact tests for categorical variables and Student’s t-tests or Wilcoxon rank-sum tests for continuous variables, as appropriate.

Liver waitlist removal status, removal reason, and timing of liver waitlist status changes were summarized descriptively to characterize the RLD-KAT cohort and were not incorporated into matching or survival models.

Survival outcomes, including death-censored graft survival, overall graft survival, and patient survival, were analyzed using the Kaplan–Meier method, with differences assessed using the log-rank test. Follow-up time was calculated from the date of kidney transplantation to the date of the outcome event or the last known follow-up.

To account for residual confounding, multivariable Cox proportional hazards models were constructed for each survival endpoint. Covariates included a priori–selected recipient (age, sex, ethnicity, BMI, diabetes, dialysis duration) and donor factors (donor age and donor type). Hazard ratios (HRs) with 95% confidence intervals (CIs) were reported. The proportional hazards assumption was evaluated using Schoenfeld residuals and visual inspection of scaled Schoenfeld plots; no major violations were detected. Missing data were minimal and handled using complete-case analysis. Statistical significance was defined as a two-sided p-value <0.05. All analyses were performed in R v4.4.1 [17].

## Results

### Baseline characteristics

Table 1 summarizes the characteristics of the RLD-KAT cohort and matched KAT controls. The mean age of both groups was identical (56.0 ± 10.3 years), and the matching strategy resulted in comparable sex distributions. Racial composition was also similar with the RLD-KAT group being predominantly white (64.4%), while the KAT group was slightly more racially diverse (59.8% white, p = 0.306). Comorbidity profiles were generally similar, including comparable rates of diabetes (34.9% in KAT vs. 35.2% in RLD-KAT). Hepatitis C was more common among RLD-KAT recipients (32.7% vs. 5.4%), reflecting the underlying liver disease history.

**TABLE 1:** Baseline recipient characteristics by transplant type.

Characteristic	Missingness (%)	KAT (n = 815)	RLD-KAT (n = 163)	*p*-value
Recipient characteristics				
Age, years	0	56.0 (10.3)	56.0 (10.3)	0.99
Female sex, n (%)	0	325 (39.9%)	66 (40.5%)	0.88
Race/Ethnicity, n (%)	0	​	​	0.31
Black	​	259 (31.8%)	42 (25.8%)	​
Other	​	69 (8.5%)	16 (9.8%)	​
White	​	487 (59.8%)	105 (64.4%)	​
BMI, kg/m^2^	0	28.5 (5.3)	26.9 (5.05)	**<0.001**
Diabetes, n (%)	0.8	282 (34.9%)	57 (35.2%)	0.94
Hepatitis B positive, n (%)	1.5	14 (1.7%)	10 (6.3%)	**<0.001**
Hepatitis C positive, n (%)	2.2	43 (5.4%)	52 (32.7%)	**<0.001**
ESRD etiology	0.2	​	​	**0.035**
Diabetes	​	292 (29.9%)	40 (24.5%)	​
Glomerulonephritis	​	174 (17.8%)	21 (12.9%)	​
Polycystic kidney disease	​	113 (11.6%)	23 (14.1%)	​
Other	​	397 (40.7%)	79 (28.5%)	​
On dialysis, n (%)	0.3	669 (82.4%)	147 (90.2%)	**0.014**
Dialysis duration, years	0.7	3.4 (3.3)	5.1 (4.2)	**<0.001**
Kidney waitlist duration, years	0	1.9 (2.1)	2.6 (2.6)	**0.001**
Peripheral arterial disease	0.9	92 (11.4%)	25 (15.3%)	0.16
Donor characteristics				
Age, years	0	43.0 (14.7)	40.6 (13.2)	0.052
Female sex, n (%)	0	377 (46.3%)	80 (49.1%)	0.51
BMI, kg/m^2^	0.5	28.1 (6.8)	28.8 (7.3)	0.20
Donation type	0	​	​	0.022
DBD	​	415 (50.9%)	99 (60.7%)	​
DCD	​	169 (20.7%)	34 (20.9%)	​
Living donor	​	231 (28.3%)	30 (18.4%)	​
Estimated GFR	27.2	85.9 (41.1)	87.8 (36.6)	0.63
KDRI	28.8	1.23 (0.30)	1.19 (0.29)	0.11
Transplant characteristics				
Local organ, n (%)	0	351 (43.1%)	87 (53.4%)	0.016
Kidney cold ischemia time, h	1.6	14.4 (10.4)	15.5 (9.6)	0.25
HLA mismatch, n (%)	0.4	​	​	0.98
0	​	47 (5.8%)	10 (6.2%)	​
1–3	​	175 (21.6%)	35 (21.6%)	​
4–6	​	590 (72.7%)	117 (72.2%)	​
Induction therapy, n (%)	7.6	​	​	0.034
Depletional	​	576 (76.3%)	96 (64.9%)	​
Non-depletional	​	137 (18.1%)	41 (27.7%)	​
Mixed induction	​	27 (3.6%)	7 (4.7%)	​
Steroids only	​	15 (2.0%)	4 (2.7%)	​
Steroid maintenance, n (%)	1.2	559 (69.4%)	111 (68.9%)	0.901
Liver disease characteristics (RLD-KAT only)				
MELD score	0	​	20.5 (4.4)	NA
INR	0	​	1.13 (0.24)	N/A
Ascites	0	​	63 (38.9%)	N/A
Encephalopathy	0	​	34 (21.0%)	N/A
TIPS, n (%)	4.3	​	5 (3.1%)	NA
Etiology of liver disease, n (%)	0	​	​	NA
Alcohol	​	​	33 (20.2%)	​
MASH	​	​	10 (6.1%)	​
Cystic/Other	​	​	49 (30.1%)	​
Autoimmune/Cholestatic (AIH, PSC, PBC)	​	​	6 (3.7%)	​
Hepatitis C	​	​	49 (30.1%)	​
Other (cryptogenic, metabolic, malignancy)	​	​	16 (9.8%)	​
Liver waitlist characteristics (RLD-KAT only)				
Liver waitlist duration, years	1.2	​	2.97 (2.56)	NA
Time from liver waitlist inactivation or removal	19	​	2.11 (2.0)	NA
Removal cause (improved)	1.2	​	105 (65.2%)	NA

Values are presented as mean (SD) or number (%), unless otherwise specified. Liver disease and liver waitlist characteristics are reported only for the RLD-KAT cohort and are presented to characterize the clinical trajectory of recipients previously listed for simultaneous liver–kidney transplantation, rather than to define cohort eligibility.

Abbreviations: AIH, Autoimmune hepatitis; BMI, Body mass index; ESRD, Human leukocyte antigen; HLA, End Stage Renal Disease; KAT, Kidney-alone transplantation; LD, Living donor; MASH, Metabolic dysfunction–associated steatohepatitis; MELD, Model for End-Stage Liver Disease; PBC, Primary biliary cholangitis; PSC, Primary sclerosing cholangitis; RLD-KAT, Recompensated liver disease kidney-alone transplantation; TIPS, Transjugular intrahepatic portosystemic shunt. Bold values denote statistically significant differences between groups (p < 0.05).

**FIGURE 1:**
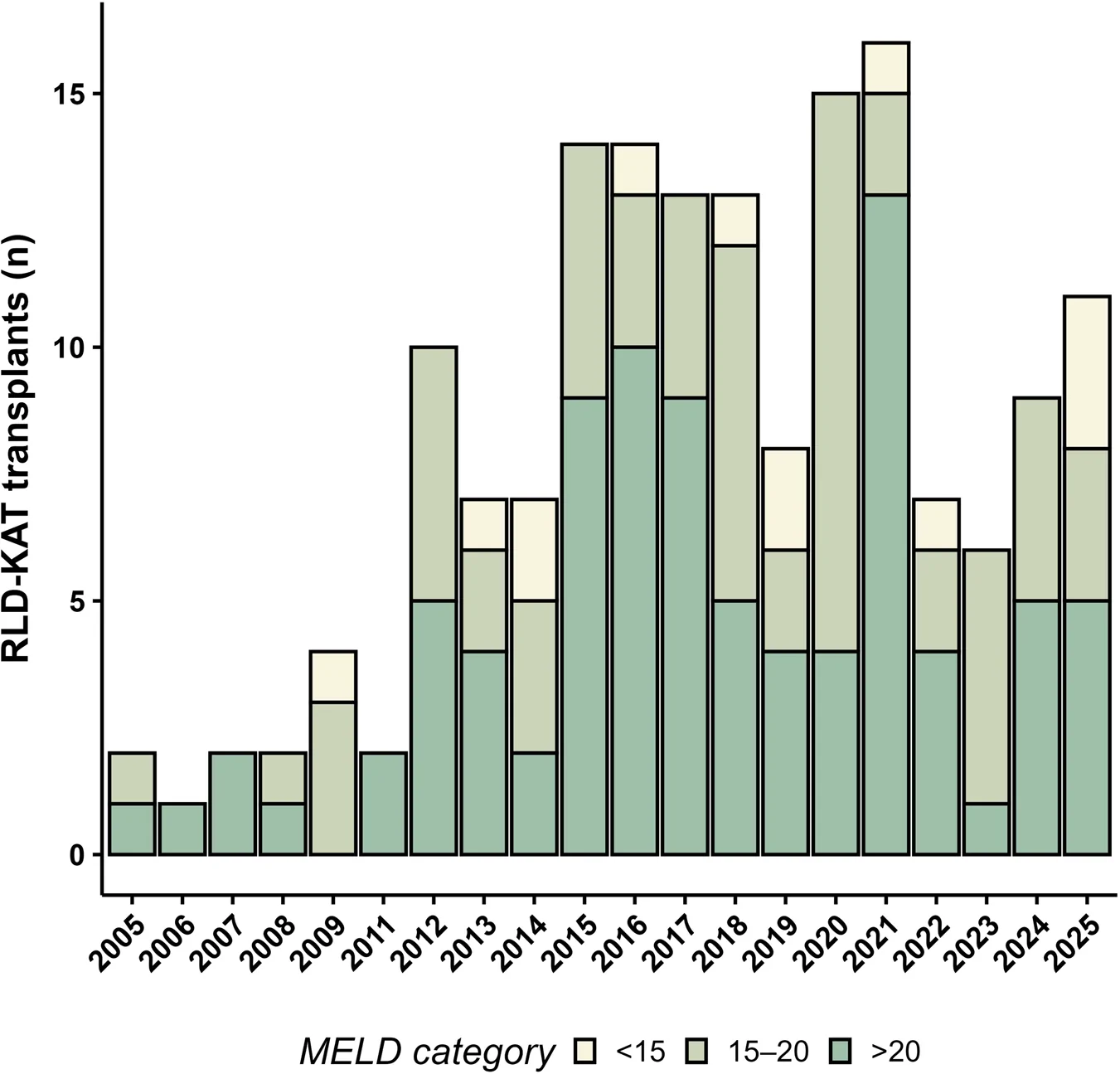
Temporal trends in kidney-alone transplantation following SLK delisting. Annual number of kidney-alone transplants among recipients previously listed for simultaneous liver–kidney transplantation and subsequently delisted (RLD-KAT), stratified by MELD score categories at the time of listing.

RLD-KAT recipients had a lower body mass index (26.9 vs. 28.5 kg/m^2^, p < 0.001), they were more likely to be on dialysis at the time of transplantation (90.2% vs. 82.4%, p = 0.014), with longer dialysis duration (5.1 vs. 3.4 years, p < 0.001) and longer waitlist time (2.6 vs. 1.9 years, p = 0.001). Among RLD-KAT recipients, the mean MELD score at initial SLK listing averaged 20.5 ± 4.4, with last mean INR of 1.13 (0.24), documented ascites in 38.9%, encephalopathy in 21.0% and 5 patients (3.1%) had previous TIPS placement at the time of registration. Cystic disease (30.1%), Hepatitis C (30.1%) and alcohol-associated liver disease (20.2%) were the leading etiologies for initial SLK listing among RLD-KAT recipients. Liver waitlist removal due to clinical improvement was documented in 105 recipients (65.2%). The mean interval from liver waitlist inactivation or removal to kidney transplantation was 2.11 ± 2.0 years. Temporal trends in RLD-KAT over the study period are shown Figure 1.

Donor characteristics were broadly comparable, although RLD-KAT recipients were less likely to receive living donor kidneys (18.4% vs. 28.3%) and more likely to receive donation after brain death organs (60.7% vs. 50.9%, p = 0.022). Donor kidney quality, as assessed by estimated GFR and KDRI, was similar between groups.

### Primary outcomes

**FIGURE 2:**
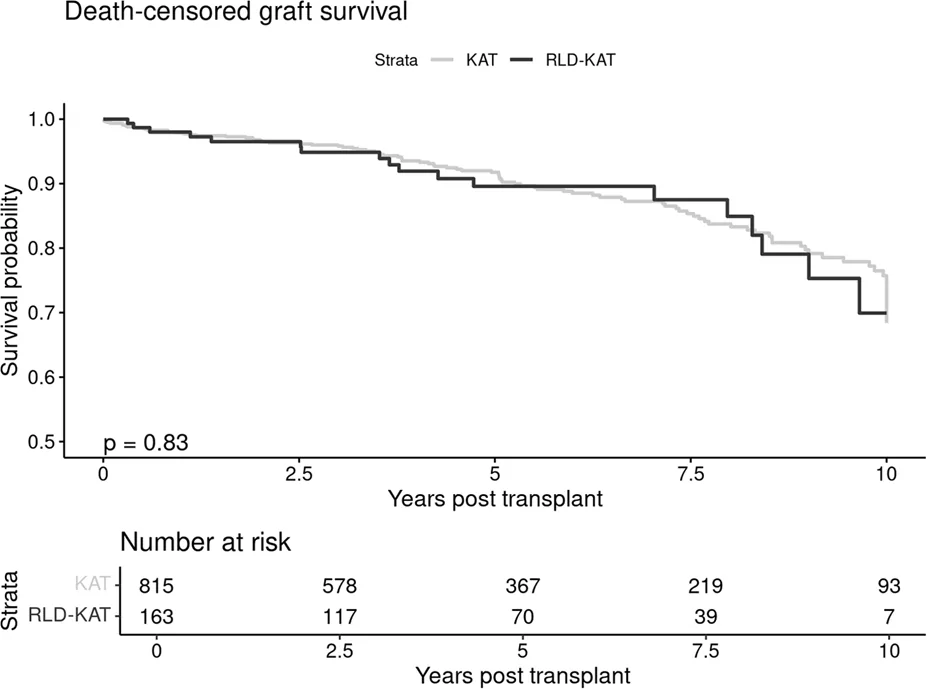
Death-censored graft survival Kaplan–Meier curves comparing death-censored graft survival between RLD-KAT recipients and matched KAT controls. No significant differences were observed between groups (log-rank p > 0.05).

Outcomes following KAT and RLD KAT are reported in Table 2. Kaplan–Meier analysis (Figure 2) did not show a significant difference in death-censored graft survival between RLD-KAT and matched KAT recipients (log-rank p = 0.830). In multivariable Cox regression adjusting for recipient demographics, diabetes, BMI, dialysis duration and donor factors, RLD-KAT was not associated with inferior graft outcomes (HR 0.89; 95% CI 0.51–1.53; p = 0.670).

**TABLE 2:** Multivariable models for recipient and kidney graft survival.

Model	HR	95% C.I.	p-value
Death-censored kidney graft survival			
Kidney alone transplant	Reference		​
RLD-KAT	0.89	(0.51, 1.53)	0.67
Overall kidney graft survival			
Kidney alone transplant	Reference		​
RLD-KAT	1.00	(0.73, 1.36)	0.980
Recipient death			
Kidney alone transplant	Reference		​
RLD-KAT	1.07	(0.76, 1.5)	0.710

Models were adjusted for recipients Age, Sex, Race, BMI, Diabetes, dialysis duration, Donor age and donor type.

Abbreviations: CI, Confidence Interval; HR, Hazard Ratio; KAT, Kidney-alone transplantation; RLD-KAT, Recompensated liver disease kidney-alone transplantation.

**FIGURE 3:**
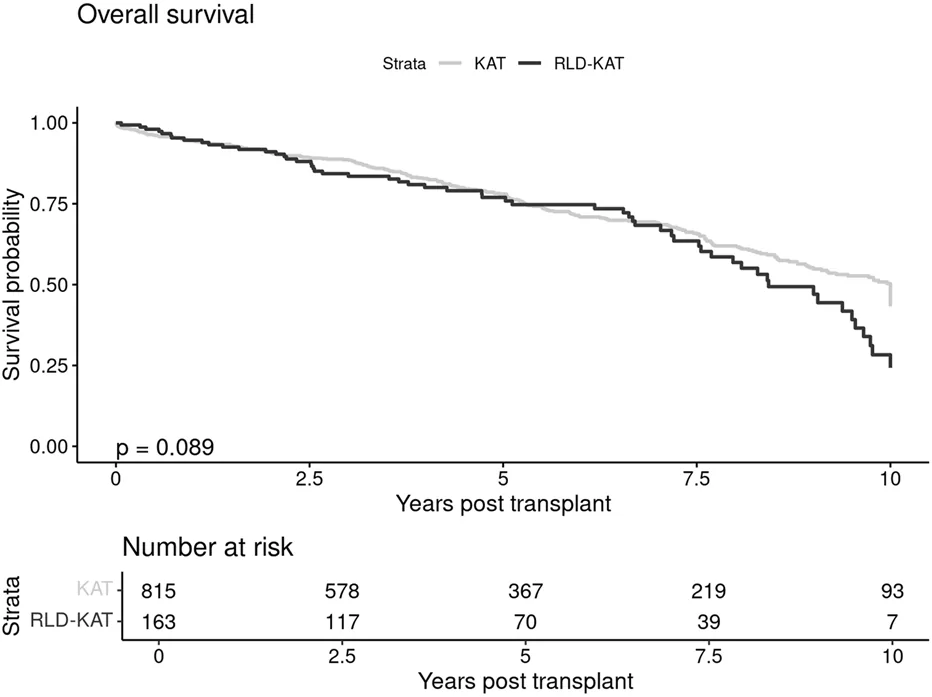
Overall graft survival. Kaplan–Meier curves demonstrating overall graft survival for RLD-KAT versus matched KAT recipients. Survival curves were similar across groups (log-rank p > 0.05).

Overall graft survival trajectories were likewise similar between groups (Figure 3; log-rank p = 0.089). After adjustment, the hazard ratio for overall graft loss among RLD-KAT recipients (HR 1.00; 95% CI 0.73–1.36; p = 0.980) did not differ significantly from matched KAT recipients.

**FIGURE 4:**
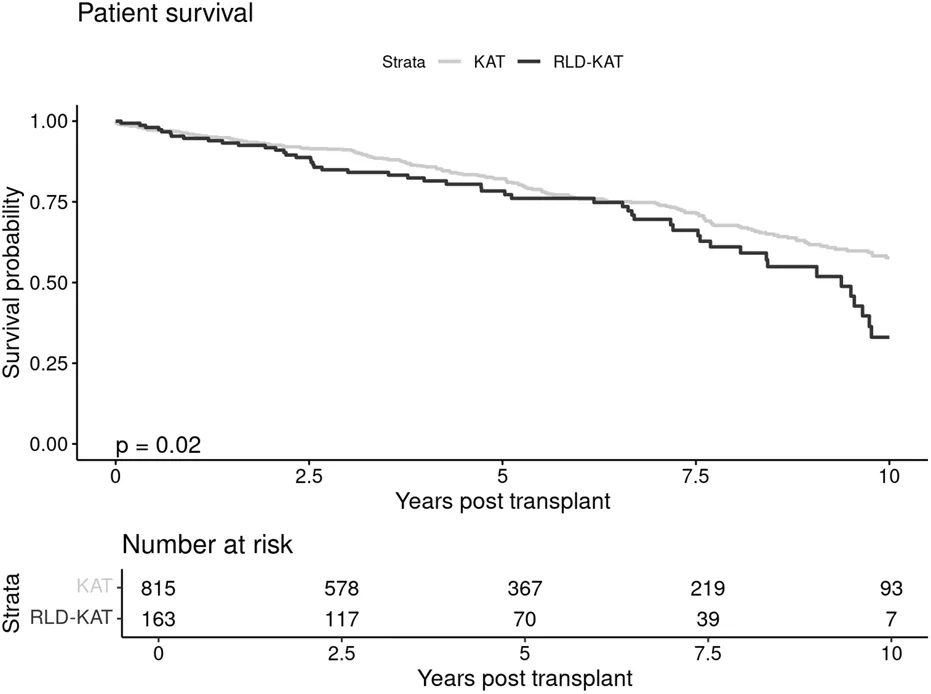
Overall patient survival. Kaplan–Meier curves for overall patient survival following transplantation in RLD-KAT and matched KAT cohorts. A statistically significant difference was observed, with slightly lower survival in the RLD-KAT group (log-rank p = 0.02).

Recipient survival was lower in RLD-KAT than controls (Figure 4; log-rank p = 0.020). However, in the adjusted model, RLD-KAT was not significantly associated with a higher risk of mortality (HR 1.07; 95% CI 0.76–1.5; p = 0.710).

In subgroup analyses stratified by underlying liver disease etiology, patient survival after KAT did not differ between RLD-KAT and matched KAT recipients across hepatitis C, alcohol-associated liver disease, polycystic liver disease, and MASH (Supplementary Figure 1).

Two additional sensitivity analyses were performed after stratifying recipients by waitlist removal status (“improved” versus all other causes). In the improved subgroup (n = 105), Kaplan–Meier analyses demonstrated no significant differences between RLD-KAT recipients and their matched KAT counterparts in recipient survival, death-censored graft survival, or overall graft survival (all log-rank *p* > 0.05) (Supplementary Figure 2). In the remaining subgroup (n = 58), death-censored and overall graft survival also did not differ between groups (both log-rank *p* > 0.05). Although unadjusted recipient survival was lower among RLD-KAT recipients (log-rank *p* = 0.005), this association was attenuated after multivariable adjustment and was no longer statistically significant (HR 1.58, 95% CI 0.89–2.79; *p* = 0.12), consistent with the findings of the primary analysis (Supplementary Figure 3).

### Secondary outcomes

Re-transplantation was rare in both groups (Table 3). Only seven (4.3%) RLD-KAT recipients required additional organ transplantation: one underwent repeat kidney transplantation, four received a subsequent liver transplant, and two ultimately required a combined SLK. These results suggest that progression to subsequent liver transplantation following kidney alone-transplantation was uncommon in this selected cohort.

**TABLE 3:** Subsequent organ transplant by recipient group.

Characteristic	KAT (n = 815)	RLD-KAT (n = 163)
Not re-transplanted	795	156
Kidney transplant alone	20	1
Liver transplant alone	0	4
Simultaneous liver/Kidney transplant	0	2

Abbreviations: KAT, Kidney-alone transplantation; RLD-KAT, Recompensated liver disease kidney-alone transplantation.

Additionally, we examined the causes of death among decedents in the full cohort. Cause-of-death information was unavailable for a substantial proportion of decedents, limiting formal comparisons between groups. Among recipients with available data, the distribution of cause-specific mortality was similar between RLD-KAT and matched KAT recipients (Supplementary Table 1).

## Discussion

In this national registry analysis, we evaluated outcomes of kidney-alone transplantation among recipients who were initially listed for SLK but ultimately underwent kidney-alone transplantation after liver transplantation was no longer pursued. These findings demonstrate that graft and patient survival in this administratively defined cohort are comparable to those of demographically matched primary KAT recipients. Across death-censored graft survival and overall graft survival, neither Kaplan–Meier analyses nor multivariable Cox models revealed significant differences between groups. While unadjusted Kaplan–Meier curves demonstrated lower patient survival in RLD-KAT recipients, this difference was attenuated and no longer statistically significant after multivariable adjustment. These findings suggest that kidney-alone transplantation may be an appropriate strategy in carefully selected prior SLK candidates following liver waitlist delisting.

The separation observed in the unadjusted Kaplan–Meier survival curve likely reflects baseline differences between groups. Adjustment for clinically relevant variables attenuated the mortality signal, suggesting that the crude survival difference was driven by measured confounders rather than an independent effect of prior liver-kidney listing status. Given the modest sample size and the inherent selection of recipients in whom liver transplantations was no longer considered necessary, these findings should not be interpreted as demonstrating equivalence but rather as supportive of acceptable outcomes in a selected cohort.

The findings of this study align with real-world clinical practice within transplant hepatology and transplant surgery. Liver transplant teams may opt to discontinue pursuit of liver transplantation when hepatic recovery is sufficient, especially when synthetic function improves, portal hypertension stabilizes, MELD components normalize, or patients respond to interventions such as alcohol abstinence, antiviral therapy, or medical optimization [18–21]. In our cohort, 65.2% of recipients were removed from the liver waitlist because of documented clinical improvement. Although the SRTR does not capture the specific clinical factors underlying these decisions, our findings demonstrate that recipients who ultimately underwent kidney-alone transplantation after liver transplantation was no longer pursued had long-term outcomes comparable to standard KAT recipients. Notably, these outcomes were observed despite the RLD-KAT group having longer pre-transplant dialysis duration and a lower proportion of living donors (Table 1). Future work should aim to establish objective criteria, such as estimation of portal hypertension, fibrosis regression markers or biochemical recovery thresholds, that can better identify individuals who can safely transition from SLK consideration to KAT.

Recent work by Schmidt et al. further supports the concept of dynamic liver transplant candidacy. In a national SRTR analysis of more than 15,000 SLK-listed candidates, prior TIPS placement was associated with significantly lower one-year mortality and a 29% reduction in adjusted mortality overall [16]. These findings emphasize the importance of considering the evolving trajectory of hepatic candidacy, when determining whether SLK or KAT is the more appropriate strategy. Our results add a valuable post-transplant perspective to this evolving paradigm. Among carefully selected SLK candidate in whom liver transplantation was not pursued, long-term outcomes were comparable to those observed among matched KAT recipients. A notable finding is the durability of this treatment strategy. Only six patients (3.7%) in the RLD-KAT cohort required subsequent liver transplantation, four liver alone transplants and two SLK, suggesting that progression to liver transplantation after KAT was uncommon. Together, these findings support a more individualized approach to SLK vs. KAT decision-making. Transplant nephrologists and surgeons should consider KAT in carefully selected prior SLK candidates, as this approach appears both clinically safe and resource efficient.

Several smaller single center studies have demonstrated that kidney transplantation alone can be safe and effective in patients with well-compensated cirrhosis [13–15, 22]. For example, Nathani et al. reported favorable post-KAT outcomes in cirrhotic patients with preserved hepatic function in a single center study including 32 patients, with 19% of cirrhotic patients presenting with hepatic decompensation post KAT. In a multicenter retrospective study including 34 patients with compensated cirrhosis, Dodge et al. demonstrated that kidney transplantation alone is feasible and associated with a five-year post-KTA incidence of hepatic decompensation of 23.5%, consistent with the natural history of disease progression in cirrhosis [23–25]. Our data complement and extend these findings by providing a national, matched cohort analysis with long-term follow-up. However, caution is warranted when generalizing our findings to all patients with compensated cirrhosis, as the RLD-KAT cohort represents a highly selected group of prior SLK candidates, and unmeasured factors such as degree of portal hypertension, presence of ascites, fibrosis, alcohol abstinence, or virologic control likely contributed to the outcomes.

A specific population warranting mention is patients with autosomal dominant polycystic kidney disease and coexisting polycystic liver disease. These individuals often present with massive hepatomegaly and progressive renal failure, and may be considered for SLK primarily to address symptom burden due to space constraints such as anorexia and abdominal distention, potentially leading to cachexia [26]. Hepatic synthetic function in this population is typically preserved, and liver disease rarely progresses to decompensation [27]. Our findings suggest that when renal dysfunction dominates the clinical picture, KAT may be an appropriate option even in patients previously listed for SLK, particularly with appropriate preoperative imaging and multidisciplinary planning (Supplementary Figure 1). Although our study was not designed specifically to evaluate this subgroup, these observations support careful patient selection and individualized decision-making regarding SLK versus KAT in polycystic liver disease.

The importance of this clinical question is likely to increase as the epidemiology and treatment of chronic liver disease continue to evolve. Alcohol-associated liver disease and MASH have become the leading indications for liver transplantation [3] and an increasing number of patients may experience meaningful improvement in liver function following sustained alcohol abstinence [18], antiviral therapy for chronic viral hepatitis [19, 20], optimization of portal hypertension [21], and emerging disease-modifying therapies for metabolic liver disease [28, 29]. As transplant candidacy becomes increasingly dynamic, multidisciplinary reassessment of both liver and kidney transplant needs will be key. Future studies should focus on developing objective clinical criteria incorporating portal hypertension, liver synthetic function, and disease trajectory to better identify candidates who may safely transition from SLK consideration to kidney-alone transplantation.

Nevertheless, several limitations merit acknowledgement. First, the SRTR lacks detailed clinical granularity regarding liver disease recovery. Importantly, the exposure of interest in this study is administrative rather than physiologic. “Recompensation” in this analysis reflects transplant center listing decisions and liver waitlist status recorded within the SRTR rather than direct measures of portal hypertension, fibrosis regression, ascites history, abstinence durability, or virologic control. Furthermore, liver waitlist removal and documented reasons for removal were used to characterize the clinical pathway preceding kidney transplantation, rather than to ascertain cohort eligibility. Consequently, variability in transplant center practices regarding waitlist management may have influenced cohort characterization without affecting cohort ascertainment. As such, this study evaluates outcomes in candidates deemed stable enough that liver transplantation was not pursued, rather than the biologic reversal of cirrhosis. The findings, therefore, reflect selection practices and center-level decision-making as much as liver disease trajectory. Second, although we identified recipients who were initially listed for SLK and ultimately underwent kidney-alone transplantation, the SRTR database does not capture individuals who were referred for liver transplant evaluation but never listed. As a result, an important segment of patients with early or improving liver function is not represented. Third, despite matching and multivariable adjustments, unmeasured confounding remains possible, particularly given the extensive clinical selection and transplant center decision-making inherent to the RLD-KAT cohort. Additionally, because the SRTR does not capture the clinical rationale underlying initial SLK listing decisions, we cannot determine whether some candidates were listed conservatively before the trajectory of their liver disease became fully apparent. As disease-modifying therapies for alcohol-associated liver disease, viral hepatitis, and MASH continue to evolve, liver transplant candidacy may become increasingly dynamic, further emphasizing the need for objective criteria to identify candidates who can safely undergo kidney-alone transplantation.

Despite these limitations, the study provides valuable insights into the durability and outcomes of kidney-alone transplantation in carefully selected prior SLK candidates after liver transplantation was no longer pursued.

## Conclusion

Kidney-alone transplantation in carefully selected candidates previously listed for SLK in whom liver transplantation was no longer pursued is associated with acceptable graft and patient survival and a low incidence of subsequent liver transplantation. These findings support careful individualized consideration of KAT over SLK in appropriately selected patients but should not be extrapolated to all patients with compensated cirrhosis or used to redefine SLK eligibility criteria. Rather, they provide reassurance regarding outcomes in this administratively defined, highly selected population.

## Data Availability

The data analyzed in this study is subject to the following licenses/restrictions: Dataset not publicly available. Requests to access these datasets should be directed to srtr@srtr.org.

## Supplementary material

SUPPLEMENTARY FIGURE 1 Patient survival after kidney-alone transplantation stratified by underlying liver disease etiology Kaplan–Meier estimates of patient survival following kidney-alone transplantation (KAT) comparing recipients without liver disease (KAT, black) and recipients with recompensated liver disease initially listed for simultaneous liver–kidney transplantation (RLD-KAT, gray). Survival is shown stratified by underlying liver disease etiology for the RLD-KAT group, including hepatitis C, alcohol-associated liver disease, polycystic liver disease, and metabolic dysfunction–associated steatohepatitis (MASH). No statistically significant differences in survival were observed between cohorts across all etiologies (log-rank p values shown).

SUPPLEMENTARY FIGURE 2 Kaplan-Meier curves for recipients removed because of clinical improvement. Kaplan–Meier curves comparing death-censored graft survival (top), overall graft survival (middle), and recipient survival (bottom) between RLD-KAT recipients with documented liver waitlist removal due to clinical improvement (n = 105) and their matched KAT controls. No significant differences were observed for death-censored graft survival, overall graft survival, or recipient survival (all log-rank p > 0.05), consistent with the primary analysis.

SUPPLEMENTARY FIGURE 3 Kaplan–Meier curves for recipients removed because of other reasons. Kaplan–Meier curves comparing death-censored graft survival (top), overall graft survival (middle), and recipient survival (bottom) between RLD-KAT recipients removed from the liver waitlist for reasons other than documented clinical improvement (n = 58) and their matched KAT controls. Death-censored graft survival and overall graft survival did not differ significantly between groups (both log-rank p > 0.05). Although unadjusted recipient survival was lower among RLD-KAT recipients (log-rank p = 0.005), this association was attenuated after multivariable adjustment and was no longer statistically significant (HR 1.58, 95% CI 0.89–2.79; p = 0.12), consistent with the primary analysis.
