# Supplementary material for: Kidney-alone transplantation in prior SLK candidates: a national registry analysis
Source: Transpl Int. 2026 Aug 27;39:16834. doi: 10.3389/ti.2026.16834 (PMC13558125; doi:10.3389/ti.2026.16834)
Supplement: Supplementary file 1 [file DataSheet1.pdf]

Kidney-Alone Transplantation in Prior SLK Candidates: A National Registry Analysis

Beat Moeckli, Kevin B. Harris, Byron Smith, David Pereyra, Jody Olson, Alexander R. Cortez, Timucin Taner; Julie K. Heimbach, Douglas A. Simonetto, Samy M. Riad

Table of contents

Supplementary Figure 1 – Patient survival stratified by underlying liver disease etiology 2

Supplementary Figure 2 – Kaplan-Meier curves for recipients removed because of clinical improvement. 3

Supplementary Figure 3 – Kaplan-Meier curves for recipients removed because of other reasons............ 4

Supplementary Figure 1 – Patient survival stratified by underlying liver disease etiology

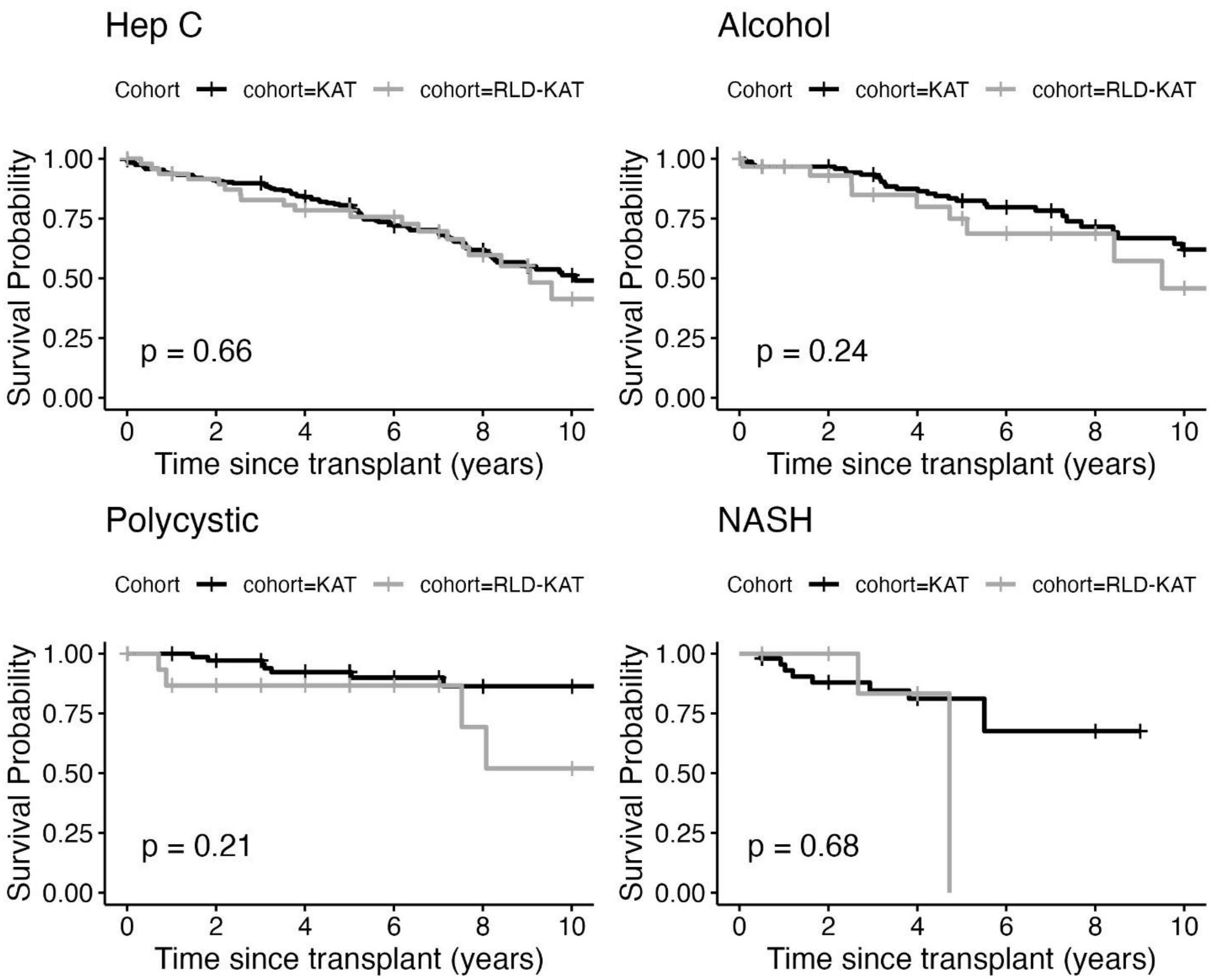

Supp. Fig. 1. Patient survival after kidney-alone transplantation stratified by underlying liver disease etiology Kaplan–Meier estimates of patient survival following kidney-alone transplantation (KAT) comparing recipients without liver disease (KAT, black) and recipients with recompensated liver disease initially listed for simultaneous liver–kidney transplantation (RLD-KAT, gray). Survival is shown stratified by underlying liver disease etiology for the RLD-KAT group, including hepatitis C, alcohol-associated liver disease, polycystic liver disease, and metabolic dysfunction–associated steatohepatitis (MASH). No statistically significant differences in survival were observed between cohorts across all etiologies (log-rank p values shown).

Supplementary Figure 2 – Kaplan-Meier curves for recipients removed because of clinical improvement.

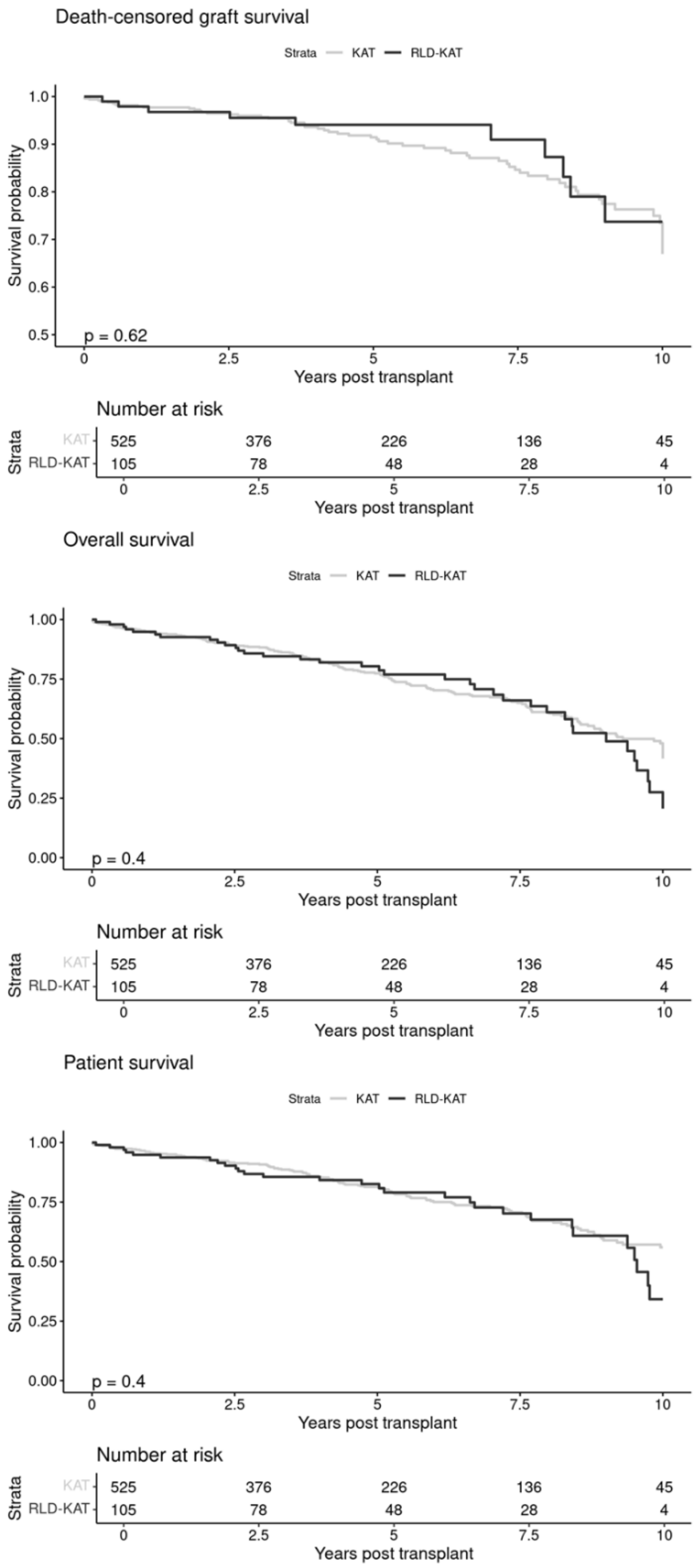

Supp. Fig. 2. Kaplan-Meier curves for recipients removed because of clinical improvement. Kaplan–Meier curves comparing death-censored graft survival (top), overall graft survival (middle), and recipient survival (bottom) between RLD-KAT recipients with documented liver waitlist removal due to clinical improvement (n = 105) and their matched KAT controls. No significant differences were observed for death-censored graft survival, overall graft survival, or recipient survival (all log-rank $p > 0.05$), consistent with the primary analysis.

Supplementary Figure 3 – Kaplan-Meier curves for recipients removed because of other reasons

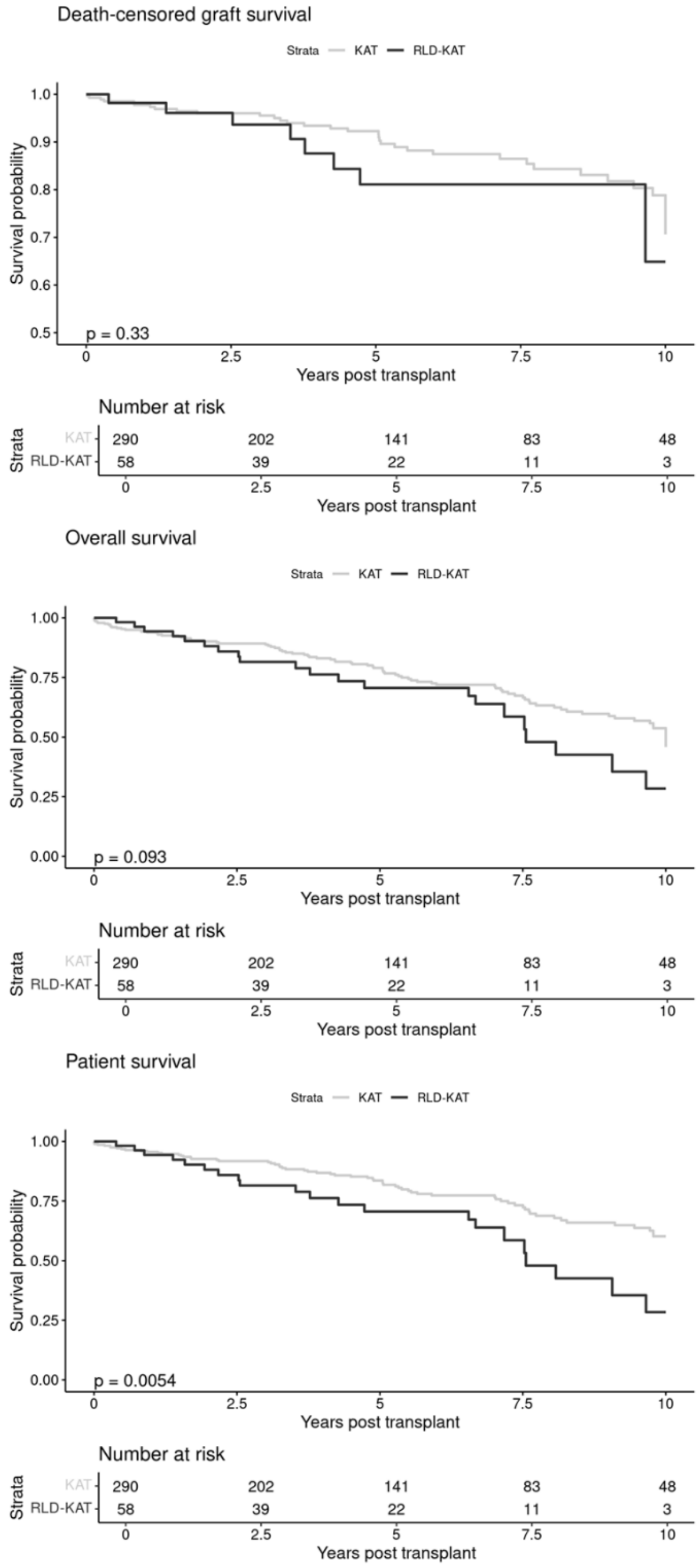

Supp. Fig. 3. Kaplan–Meier curves for recipients removed because of other reasons
Kaplan–Meier curves comparing death-censored graft survival (top), overall graft survival (middle), and recipient survival (bottom) between RLD-KAT recipients removed from the liver waitlist for reasons other than documented clinical improvement (n = 58) and their matched KAT controls. Death-censored graft survival and overall graft survival did not differ significantly between groups (both log-rank $p > 0.05$). Although unadjusted recipient survival was lower among RLD-KAT recipients (log-rank $p = 0.005$), this association was attenuated after multivariable adjustment and was no longer statistically significant (HR 1.58, 95% CI 0.89–2.79; $p = 0.12$), consistent with the primary analysis.
